# Unique Neural Mechanisms Underlying Speed Control of Low-Force Ballistic Contractions

**DOI:** 10.5114/jhk/182889

**Published:** 2024-02-02

**Authors:** Joongsuk J. Kim, Stefan Delmas, Yoon Jin Choi, Jessica C. Hubbard, Michelle Weintraub, Fotini Arabatzi, Basma Yacoubi, Evangelos A. Christou

**Affiliations:** 1 Department of Applied Physiology and Kinesiology, University of Florida, Gainesville, FL, USA.; 2 School of Physical Education and Sports Science (Serres), Aristotle University of Thessaloniki, Serres, Greece.; 3 Department of Neurology, Norman Fixel Institute of Neurological Disorders, University of Florida.Gainesville, FL, USA.

**Keywords:** time to peak force, rate of force development, EMG, speed-control hypothesis, neural oscillation

## Abstract

According to the speed-control hypothesis, the rate of force development (RFD) during ballistic contractions is dictated by force amplitude because time to peak force (TPF) remains constant regardless of changes in force amplitude. However, this hypothesis has not been tested at force levels below 20% of an individual’s maximum voluntary contraction (MVC). Here, we examined the relationship between the RFD and force amplitude from 2 to 85% MVC and the underlying structure of muscle activity in 18 young adults. Participants exerted ballistic index finger abductions for 50 trials in each of seven randomly assigned force levels (2, 5, 15, 30, 50, 70, and 85% MVC). We quantified TPF, RFD, and various EMG burst characteristics. Contrary to the speed-control hypothesis, we found that TPF was not constant, but significantly varied from 2 to 85% MVC. Specifically, the RFD slope from 2 to 15% MVC was greater than the RFD slope from 30 to 85% MVC. Longer TPF at low force levels was associated with the variability of EMG burst duration, whereas longer TPF with higher force levels was associated with the EMG burst integral. Contrary to the speed-control hypothesis, we found that the regulation of TPF for low and high force levels was different, suggesting that neuronal variability is critical for force levels below 30% MVC and neuronal amplitude for force levels above 30% MVC. These findings present compelling new evidence highlighting the limitations of the speed-control hypothesis underscoring the need for a new theoretical framework.

## Introduction

Voluntary contractions performed with maximal velocity and short muscle contraction times are termed ballistic contractions (Desmedt and Godaux, 1977; Freund, 1983; Zehr and Sale, 1994). The current belief, originated by Freund and Büdingen (1978), is that the rate of force development (RFD; force amplitude with respect to time) during ballistic contractions is primarily affected by the force amplitude. The RFD is quantified as the slope of the force-time curve, which shows the contraction speed from the force onset to peak force (Freund and Büdingen, 1978; Maffiuletti et al., 2016; Mirkov et al., 2004). It has been shown that duration from the force onset to peak force (termed time to peak force; TPF) remains constant with changes in force amplitude during ballistic contractions, resulting in a linear relationship between the RFD and force amplitude (Freund and Büdingen, 1978; Maffiuletti et al., 2016; Mirkov et al., 2004). According to the speed-control hypothesis, TPF and electromyography (EMG) burst duration remain approximately constant during ballistic contractions across varying force levels (Freund and Büdingen, 1978). This suggests that the central nervous system regulates the amplitude of force during ballistic contractions to control the speed of force generation. Based on their findings, Freund and Büdingen (1978) proposed the speed-control hypothesis, which has been tested and supported by other studies (Bellumori et al., 2011; Boccia et al., 2018; Enoka, 1983; Kozinc et al., 2022). A significant limitation of all these studies, however, is the omission of contraction force levels below 20% of maximum. Thus, the relationship between the RFD and the whole range of force levels during ballistic contractions remains unclear. Here, we tested the speed-control hypothesis with ballistic contractions ranging in force levels from 2 to 85% MVC and examined the underlying muscle activity of these ballistic contractions.

Over the last 30 years, the primary interest has been the muscle activity which regulates TPF and the RFD at different force levels (Aagaard et al., 2002; Haff et al., 1997; Kozinc et al., 2022; Paasuke et al., 2001). There is evidence that ballistic contractions with a greater RFD have a faster discharge rate of motor units and very brief interspike intervals (<10 ms) in the initial discharges (Desmedt and Godaux, 1977; Duchateau and Baudry, 2014; Miles et al., 2005; Van Cutsem and Duchateau, 2005). There is also evidence that the RFD of ballistic contractions relates to neural oscillations. For example, a greater RFD is associated with greater whole muscle activity oscillations from 30 to 60 Hz (Alegre et al., 2004; Joundi et al., 2012) and greater cortical oscillations from 30 to 60 Hz assessed with electroencephalographic recording (EEG) methods (Crone et al., 1998; Engel and Fries, 2010; Jensen and Mazaheri, 2010). Although we know that a greater RFD is associated with an altered neural drive to the motor neuron pool, we are currently unaware of how muscle activity explains the RFD at low force levels.

In this study, we examined the relationship between the RFD and force levels ranging from 2 to 85% MVC using abduction of the index finger as a movement model, which is controlled by a single muscle (First Dorsal Interosseous muscle; FDI). This experimental paradigm, therefore, allowed us to better study the underlying muscle activity that generates the RFD at each force level. We hypothesized that both TPF and RFD would show distinct profiles at low force levels and ballistic contractions at low force levels would be modulated by distinct neuromuscular activity.

## Methods

### Participants

Eighteen young adults (26.3 ± 6.4 years, 9 women) volunteered to participate in this study. All participants reported being healthy with no evidence of neurological or musculoskeletal disease. Participants were all right-handed in accordance with the Edinburgh Handedness Inventory (Oldfield, 1971). The Institutional Review Board at the University of Florida approved the procedures of this study (approval code: IRB201901681; approval date: 23 July 2019), and all the participants signed an informed consent form before the initiation of the experiment.

### Measures

**Figure 1:**
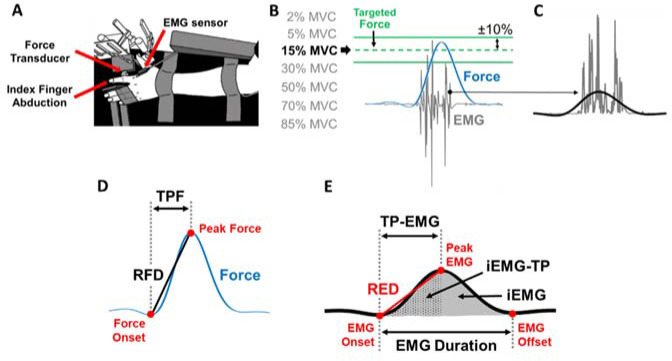
Experimental setup and the protocol for the ballistic contraction task. (A) Top view of the experimental set up for testing ballistic abduction of the left index finger. Participants performed index finger abductions as fast as possible with one EMG sensor on the FDI muscle. (B) There were two green lines on the monitor which indicated ±10% from each targeted force for each of the seven randomly assigned effort levels. (C) Rectified EMG (grey lines) and filtered EMG (solid black line) at 6 Hz with a fourth-order Butterworth filter are shown. (D) We identified Time to Peak Force (TPF), Peak Force (PF), and the Rate of Force Development (RFD) of each trial in the low-pass filtered force signal. (E) We identified Time to Peak EMG (TP-EMG), EMG duration, Peak EMG, the Rate of EMG Development (RED), integrated EMG (iEMG), and integrated EMG until TP-EMG (iEMG-TP) of each trial in the low-pass filtered EMG signal.

Each participant sat comfortably in an upright position, facing a 30-inch monitor (HP ZR30w, Hewlett-Packard Development Company, Houston, TX, USA) located 1.43 m away at the eye level. Participants flexed the left shoulder to ~45° and the left elbow to ~90°. The left forearm was pronated and immobilized by straps on a customized metal plate. Only the index finger was free to move. This arrangement allowed to measure the force of the abduction of the index finger in the horizontal plane, which was produced almost exclusively by the contraction of FDI muscle (Figure 1A). All participants performed the ballistic contraction tasks with their non-dominant hand to introduce greater novelty to the task.

The isometric force produced by the abduction of the index finger was measured by a one-dimensional force transducer (model 41, 50 lbs capacity, Honeywell Sensotec Inc., Columbus, OH, USA). The force signals were sampled at 1,000 Hz with a Power 1401 A/D board (Cambridge Electronic Design, Cambridge, UK) and a NI-DAQ card (model USB6210, National Instruments, Austin, TX, USA). The FDI muscle activity was measured by a surface EMG electrode (Trigno EMG sensor, Delsys Inc., Boston, MA, USA) that was placed in line with the muscle fibers. The EMG signals were sampled at 1,000 Hz with a Power 1401 A/D board and a NI-DAQ card.

We measured the maximal voluntary contraction (MVC) for index finger abduction force before the ballistic contraction tasks. Participants increased their index finger abduction force to their maximum as fast as possible and maintained it for three seconds. Participants performed three to five MVC trials until two MVC values were within 5% of each other. One minute of rest was given between trials.

### Design and Procedures

We instructed participants to increase their index finger abduction force within a targeted force area as fast as possible. Participants received visual feedback of their performance after each trial. There were two green lines on the monitor which indicated ±10% from each targeted force (Figure 1B) for each of seven randomly assigned effort levels (2, 5, 15, 30, 50, 70, and 85% MVC). We displayed the target area as two green lines and the force produced by the participant as a blue line. The order of the effort levels was randomized (block randomization). Participants performed 50 trials in a row (one block) for each effort level and had three-minute rest after each block. Participants performed one testing session in about two hours. Each participant performed the following procedures: 1) familiarization of the experimental procedure that included a verbal explanation and practice trials of the ballistic contraction tasks using the left index finger abduction, 2) maximal voluntary contraction (MVC) tasks with index finger abduction, and 3) 50 trials of ballistic contraction tasks at each of seven randomly assigned effort levels.

### Data Analysis

The force signals were recorded and analyzed using a custom-written program in Matlab (MathWorks, Natick, MA). EMG signals were acquired with EMG-works Software (Delsys) and analyzed using a custom-written program in Matlab. We low-pass filtered the raw force signal at 6 Hz with a fourth-order Butterworth filter. The raw EMG signal was rectified and low-pass filtered at 6 Hz with a fourth-order Butterworth filter. We quantified TPF and the RFD from the low-pass filtered force signal. The force onset was identified as the local minima in the detrended force signal before peak force (PF). TPF of each trial was identified as the duration between the force onset to PF. The RFD of each trial was calculated as PF divided by TPF (Figure 1D).

**Figure 2:**
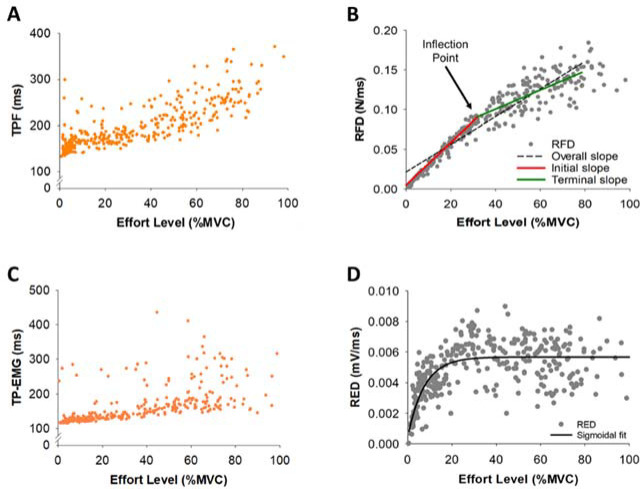
Representative example of variables for a single participant for all trials. (A) Representative example of Time to Peak Force (TPF) for a single participant for all trials (orange dots). (B) Representative example of the Rate of Force Development (RFD) for a single participant for all trials (gray dots). The inflection point (IP) for the RFD data indicates the first instance that the RFD slope slows down with effort. The RFD slope before the IP (termed initial slope; red line) and the RFD slope after the IP (termed terminal slope; green line). The RFD slope from 2 to 80% MVC was termed the overall slope (black dotted line). (C) Representative example of Time to Peak EMG (TP-EMG) for a single participant for all trials (orange dots). (D) Representative example of the Rate of EMG Development (RED) for a single participant for all trials (gray dots). We used the sigmoidal function fitting (black line) to find the corresponding trendline of RED.

We quantified Time to Peak EMG (TP-EMG), EMG Duration, the Rate of EMG Development (RED), and integrated EMG (iEMG) from the rectified and low-pass filtered EMG signal (Figure 1E). The EMG onset and offset were identified as the local minima of the filtered and detrended EMG signal before Peak EMG and after Peak EMG, respectively. TP-EMG of each trial was identified as the duration between the EMG onset to Peak EMG. EMG Duration of each trial was identified as the duration between the EMG onset to the EMG offset. The RED of each trial was calculated as Peak EMG divided by TP-EMG. We used a sigmoidal function to fit the raw data and identify the corresponding trendline of the RED (Figure 2D). iEMG of each trial was calculated as the area under the curve of the rectified and filtered EMG (Figure 1E). Additionally, integrated EMG until TP-EMG (iEMG-TP) of each trial was calculated as the area under the curve of the rectified and filtered EMG from the EMG onset to TP-EMG.

Within the RFD curve (Figure 2B), we quantified the inflection point (IP) using the 8^th^-order polynomial curve fitting to find the corresponding trendline of the RFD. We filtered the RFD under 80% MVC to minimize the drift of the trendline due to the relatively large standard deviation over 80% MVC. The first RFD slope before the IP location was termed the initial slope and the second RFD slope after the IP location was termed the terminal slope (Figure 2B). The RFD slope from 2% to 80% MVC was termed the overall slope. We noticed that there were two distinct RFD slope profiles in the initial slope, thus we performed a K-means cluster analysis for grouping participants (N = 18) into two distinct profiles to examine if the modulation of muscle activity differed for these two groups. The RFD slopes were clustered based on a common nearest mean (a cluster center). The group with slow rates for the initial RFD slope was termed Slow Initial and the group with fast rates for the initial RFD slope was termed Fast Initial (Figure 7A). A K-means cluster analysis was performed with the SPSS Statistics 27 (IBM, Armonk, NY).

**Figure 3:**
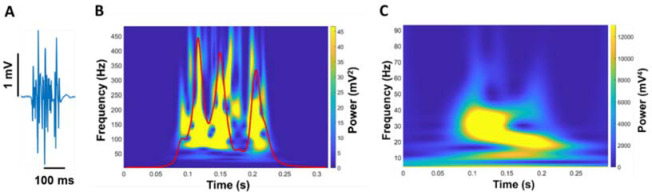
Quantification of EMG modulation. The raw EMG signal of the FDI muscle (A) was wavelet transformed (B). From the wavelet transformed EMG, we summed the power from 20 to 400 Hz with respect to frequency and the summation of power in EMG from 20 to 400 Hz was expressed in power versus time (red line in B). (C) Next, we wavelet transformed the summed signal and quantified the power from 4–8, 8–13, 13–30, 30–60, and 60–100 Hz.

**Figure 4:**
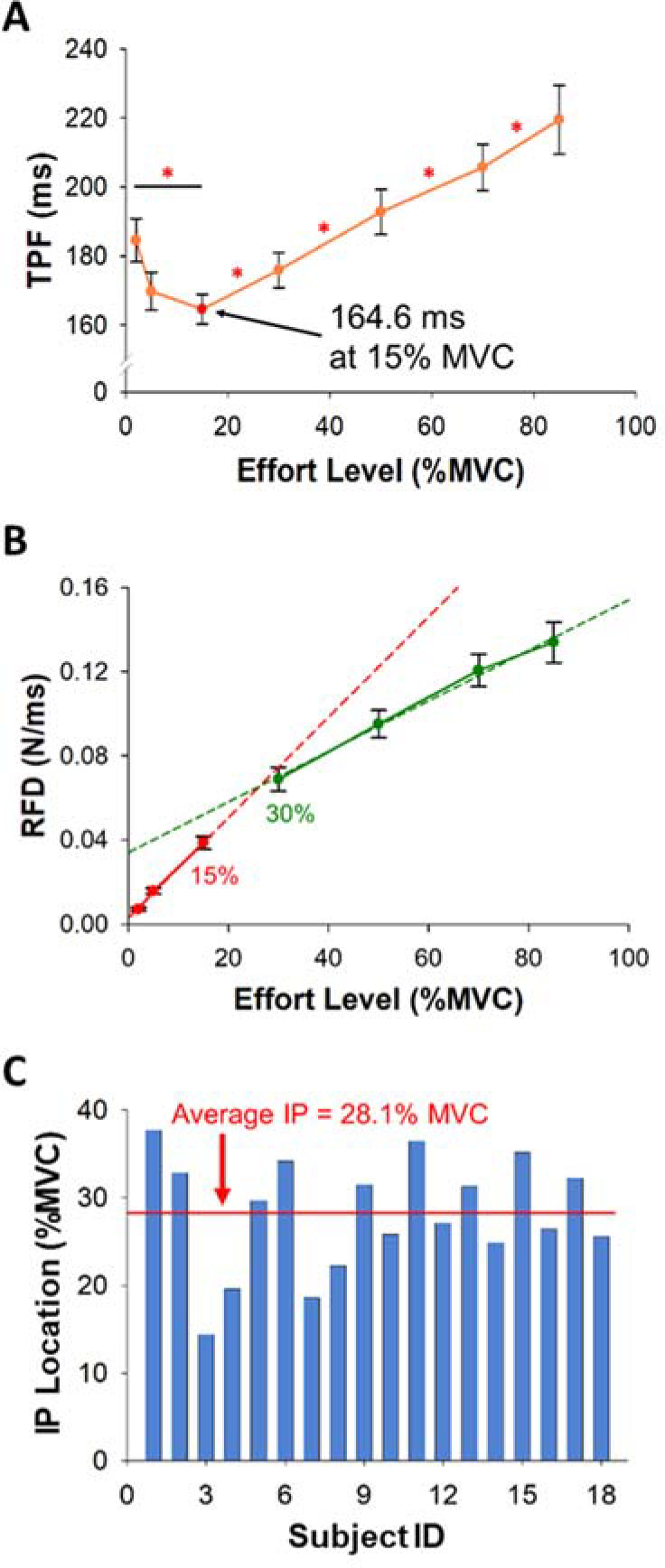
Time to Peak Force (TPF) and the Rate of Force Development (RFD) as a function of the effort level. Inflection point (IP) of each participant. (A) TPF with the effort level (%MVC) of all participants. TPF was not constant but varied with effort. The shortest TPF occurred at 15% MVC. TPF at 2% MVC was longer than TPF at 15% MVC. (B) The RFD with the effort level (%MVC) of all participants. The RFD increase was faster at the effort level below 15% MVC. (C) The average IP of all participants was 28.1% MVC (red line).

**Figure 5:**
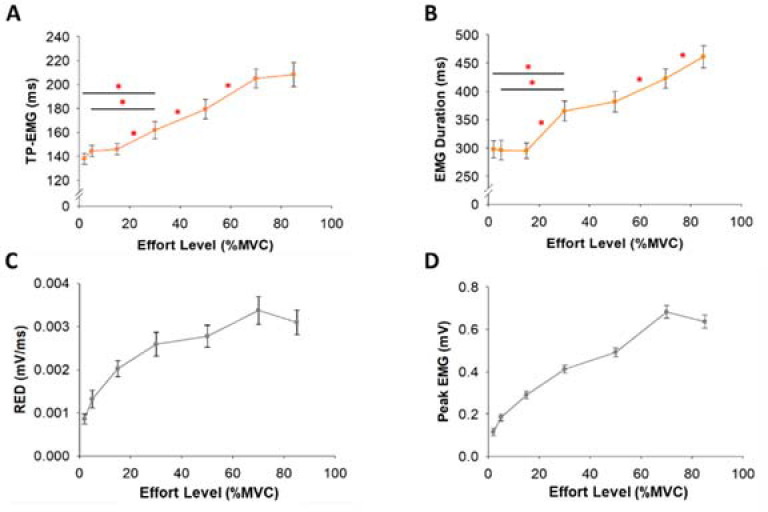
Time to Peak EMG (TP-EMG), EMG Duration, the Rate of EMG Development (RED), and Peak EMG as a function of the effort level. (A) TP-EMG with the effort level (%MVC) of all participants. (B) EMG Duration with the effort level (%MVC) of all participants was identified as the time difference between the EMG onset to the EMG offset. TP-EMG and EMG Duration were not constant but varied with effort. (C) The RED with the effort level (%MVC) of all participants. The RED was calculated as Peak EMG (D) divided by TP-EMG. The relationship between the RED and the effort level was non-linear.

**Figure 6:**
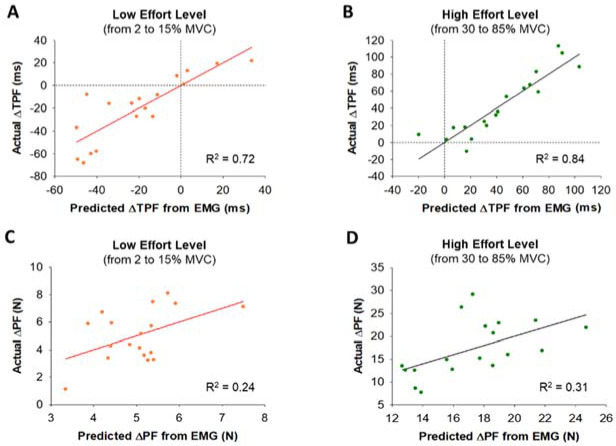
Stepwise multiple linear regression model of Time to Peak Force (TPF) and Peak Force (PF) predicted from EMG at low (from 2 to 15% MVC) and high (from 30 to 85% MVC) effort levels. (A) The lengthening in TPF at 2% MVC relative to the 15% MVC effort level was associated with Time to Peak EMG (TP-EMG) and variability (CV) of EMG Duration (R^2^ = 0.72). (B) The increase in TPF at 85% MVC relative to the 30% MVC effort level was associated with TP-EMG and integrated EMG (iEMG) (R^2^ = 0.84). (C) The increase in PF at the low effort level was associated with the variability (CV) of iEMG (R^2^ = 0.24). (D) The increase in PF at the high effort level was associated with iEMG until TP-EMG (iEMG-TP) (R^2^ = 0.31).

**Figure 7:**
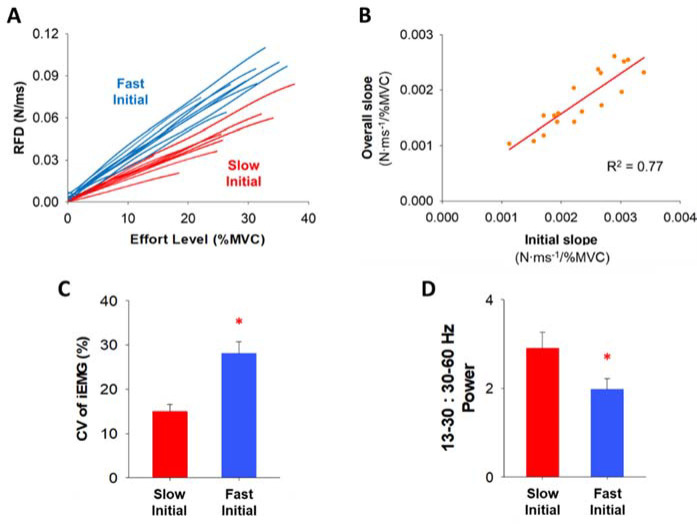
Cluster analysis within the initial slope of the RFD curve and the relationship between the initial slope and the overall slope of the RFD curve. Comparison of FDI muscle activity between two clustering groups (Slow Initial & Fast Initial) within the initial slope of the RFD curve. (A) The 18 participants were clustered into two groups (fast and slow) based on the rate of the initial slope of the RFD curve. Each group contained 9 participants (blue for fast and red for slow). (B) The overall slope of the RFD curve strongly correlated with the initial slope. It suggests that the RFD profiles at the low effort level were highly predictable index to anticipate the whole RFD profile. (C) The variability (CV) of integrated EMG (iEMG) at four low effort levels (2, 5, 15, and 30% MVC) for the participants with the slow RFD initial slope (Slow Initial) and the fast RFD initial slope (Fast Initial). Participants in the Fast Initial group exhibited significantly greater variability (CV) of iEMG. (D) We examined the normalized power of FDI EMG modulation for participants in the Slow Initial group and the Fast Initial group in five different frequency bands (4–8, 8–13, 13–30, 30–60, and 60–100 Hz) and participants in the Fast Initial group exhibited a significantly lower ratio in the normalized power of 13–30 Hz (beta) to 30–60 Hz (gamma).

We analyzed a wavelet transform from the full EMG signal to quantify the neural activity of the FDI muscle (Figure 3). A wavelet analysis can provide evidence about the relationship between motor output and EMG modulation (Neto et al., 2010). First, we cut the wavelet spectra of the full EMG signal ranging from 20 to 400 Hz and quantified an integral with respect to frequency. The wavelet integral was expressed in power versus time (EMG modulation profile). We conducted the wavelet transform from this new signal to quantify the modulation of EMG. Our focus was on the frequency ranging from 4 to 100 Hz in five different frequency bands (4–8, 8–13, 13–30, 30–60, and 60–100 Hz). To compare the power between participants, we quantified the normalized power of each frequency band relative to the total power from 4 to 100 Hz.

### Statistical Analysis

The outcomes (dependent variables) for each contraction were the mean, standard deviation, and the coefficient of variation (CV) of TPF, PF, RFD, TP-EMG, EMG Duration, Peak EMG, RED, iEMG, iEMG-TP, and EMG modulation power. The normality of the data was examined using the Kolmogorov-Smirnov test and sphericity violations were adjusted using the Greenhouse-Geisser correction. A repeated measures one-way ANOVA or the Friedman test was used to examine the differences in the outcomes between the seven levels of force amplitude (independent variable). A post-hoc analysis was conducted with the Benjamini-Hochberg procedure to control for a false discovery rate (FDR) at the level of significance, α = 0.05 (Benjamini et al., 2006). We used a stepwise multiple linear regression model to establish statistical models to predict: 1) the increase in TPF from EMG at low effort levels (from 2 to 15% MVC), 2) the increase in TPF from EMG at high effort levels (from 30 to 85% MVC), 3) the increase in PF from EMG at low effort levels, and 4) the increase in PF from EMG at high effort levels. The goodness-of-fit for the linear regression model was given by the coefficient of determination (R^2^) and Durbin-Watson statistic (DW). A K-means cluster analysis was used for grouping two distinct profiles in the initial RFD slope. A repeated measures two-way ANOVA was used to examine the differences in the EMG outcomes at four levels of low force amplitude below 30% MVC between two clustering groups. Furthermore, an independent *t*-test was used to determine the differences in the modulation of EMG in each of the frequency bands between two clustering groups. All statistical analyses were performed with the SPSS Statistics 27 (IBM, Armonk, NY). Data are presented as means ± standard errors (SE) infigures.

## Results

### TPF and RFD

TPF significantly varied (F(2,34) = 17.6, *p*< 0.001, η^2^ = 0.5) with the effort level (Figure 4A). Our most important finding was that TPF was significantly longer (*p* = 0.015; Figure 4A, Table 1) at 2% MVC (184.6 ms) than at 15% MVC (164.6 ms). The RFD varied significantly (F(2,25) = 155.2, *p*< 0.001, η^2^ = 0.9) with the effort level (Figure 4B, Table 1). We found an inflection point (IP) on the RFD curve as a function of effort. Figure 4C shows the IP location for each participant. The average IP location was 28.1% MVC. This IP demonstrates that the RFD increase with effort was slower after ~30% MVC. The RFD slope was significantly steeper (*p*< 0.001; Figure 4B) at 2–15% MVC (2.3×10^−3^ N·ms^−1^/%MVC) than at 30–85% MVC (1.5×10^−3^ N·ms^−1^/%MVC). There was no significant correlation between the IP location and FDI strength (R^2^ = 0.15, *p* = 0.12).

### EMG Burst

TP-EMG (χ^2^(6) = 83.4, *p*< 0.001, W = 0.8) and EMG Duration (χ^2^(6) = 76.2, *p*< 0.001, W = 0.7) varied significantly with the effort level (Figure 5A & 5B). Moreover, the RED (χ^2^(6) = 86.7, *p*< 0.001, W = 0.8) and Peak EMG (χ^2^(6) = 99.1, *p*< 0.001, W = 0.9) varied significantly with the effort level (Figure 5C & 5D). The statistical differences of TP-EMG, EMG Duration, RED, and Peak EMG among different effort levels are shown in Table 1.

**Table 1:** Variables as a function of effort.

%MVC	Variable	2	5	15	30	50	70	85
2	TPF	-	*p =* 0.051	*p =* 0.015*	*p =* 0.222	*p =* 0.364	*p =* 0.051	*p =* 0.020*
	RFD		*p <* 0.001*	*p <* 0.001*	*p <* 0.001*	*p <* 0.001*	*p <* 0.001*	*p <* 0.001*
	TP-EMG^a^		*p =* 0.147	*p =* 0.147	*p =* 0.001*	*p <* 0.001*	*p <* 0.001*	*p <* 0.001*
	EMG Duration^a^		*p =* 0.715	*p =* 0.811	*p =* 0.003*	*p =* 0.002*	*p <* 0.001*	*p <* 0.001*
	RED^a^		*p <* 0.001*	*p <* 0.001*	*p <* 0.001*	*p <* 0.001*	*p <* 0.001*	*p <* 0.001*
	Peak EMG^a^		*p <* 0.001*	*p <* 0.001*	*p <* 0.001*	*p <* 0.001*	*p <* 0.001*	*p <* 0.001*
5	TPF	*p =* 0.051	-	*p =* 0.148	*p =* 0.132	*p <* 0.001*	*p <* 0.001*	*p <* 0.001*
	RFD	*p <* 0.001*		*p <* 0.001*	*p <* 0.001*	*p <* 0.001*	*p <* 0.001*	*p <* 0.001*
	TP-EMG^a^	*p =* 0.147		*p =* 0.711	*p =* 0.014*	*p =* 0.001*	*p <* 0.001*	*p <* 0.001*
	EMG Duration^a^	*p =* 0.715		*p =* 0.811	*p =* 0.003*	*p <* 0.001*	*p <* 0.001*	*p <* 0.001*
	RED^a^	*p <* 0.001*		*p <* 0.001*	*p <* 0.001*	*p <* 0.001*	*p <* 0.001*	*p <* 0.001*
	Peak EMG^a^	*p <* 0.001*		*p <* 0.001*	*p <* 0.001*	*p <* 0.001*	*p <* 0.001*	*p <* 0.001*
15	TPF	*p =* 0.015*	*p =* 0.148	-	*p <* 0.001*	*p <* 0.001*	*p <* 0.001*	*p <* 0.001*
	RFD	*p <* 0.001*	*p <* 0.001*		*p <* 0.001*	*p <* 0.001*	*p <* 0.001*	*p <* 0.001*
	TP-EMG^a^	*p =* 0.147	*p =* 0.711		*p <* 0.001*	*p <* 0.001*	*p <* 0.001*	*p <* 0.001*
	EMG Duration^a^	*p =* 0.811	*p =* 0.811		*p =* 0.005*	*p <* 0.001*	*p <* 0.001*	*p <* 0.001*
	RED^a^	*p <* 0.001*	*p <* 0.001*		*p =* 0.008*	*p <* 0.001*	*p <* 0.001*	*p <* 0.001*
	Peak EMG^a^	*p <* 0.001*	*p <* 0.001*		*p <* 0.001*	*p <* 0.001*	*p <* 0.001*	*p <* 0.001*
30	TPF	*p =* 0.222	*p =* 0.132	*p <* 0.001*	-	*p <* 0.001*	*p <* 0.001*	*p <* 0.001*
	RFD	*p <* 0.001*	*p <* 0.001*	*p <* 0.001*		*p <* 0.001*	*p <* 0.001*	*p <* 0.001*
	TP-EMG^a^	*p =* 0.001*	*p =* 0.014*	*p <* 0.001*		*p =* 0.003*	*p <* 0.001*	*p <* 0.001*
	EMG Duration^a^	*p =* 0.003*	*p =* 0.003*	*p =* 0.005*		*p =* 0.232	*p =* 0.007*	*p <* 0.001*
	RED^a^	*p <* 0.001*	*p <* 0.001*	*p =* 0.008*		*p =* 0.280	*p =* 0.008*	*p =* 0.015*
	Peak EMG^a^	*p <* 0.001*	*p <* 0.001*	*p <* 0.001*		*p =* 0.011*	*p <* 0.001*	*p <* 0.001*
50	TPF	*p =* 0.364	*p <* 0.001*	*p <* 0.001*	*p <* 0.001*	-	*p =* 0.014*	*p =* 0.002*
	RFD	*p <* 0.001*	*p <* 0.001*	*p <* 0.001*	*p <* 0.001*		*p <* 0.001*	*p <* 0.001*
	TP-EMG^a^	*p <* 0.001*	*p =* 0.001*	*p <* 0.001*	*p =* 0.003*		*p =* 0.001*	*p <* 0.001*
	EMG Duration^a^	*p =* 0.002*	*p <* 0.001*	*p <* 0.001*	*p =* 0.232		*p =* 0.011*	*p =* 0.003*
	RED^a^	*p <* 0.001*	*p <* 0.001*	*p <* 0.001*	*p =* 0.280		*p =* 0.039*	*p =* 0.026*
	Peak EMG^a^	*p <* 0.001*	*p <* 0.001*	*p <* 0.001*	*p =* 0.011*		*p =* 0.002*	*p <* 0.001*
70	TPF	*p =* 0.051	*p <* 0.001*	*p <* 0.001*	*p <* 0.001*	*p =* 0.014*	-	*p =* 0.051
	RFD	*p <* 0.001*	*p <* 0.001*	*p <* 0.001*	*p <* 0.001*	*p <* 0.001*		*p =* 0.008*
	TP-EMG^a^	*p <* 0.001*	*p <* 0.001*	*p <* 0.001*	*p <* 0.001*	*p =* 0.001*		*p =* 0.496
	EMG Duration^a^	*p <* 0.001*	*p <* 0.001*	*p <* 0.001*	*p =* 0.007*	*p =* 0.011*		*p =* 0.043*
	RED^a^	*p <* 0.001*	*p <* 0.001*	*p <* 0.001*	*p =* 0.008*	*p =* 0.039*		*p =* 0.286
	Peak EMG^a^	*p <* 0.001*	*p <* 0.001*	*p <* 0.001*	*p <* 0.001*	*p =* 0.002*		*p =* 0.811
85	TPF	*p =* 0.020*	*p <* 0.001*	*p <* 0.001*	*p <* 0.001*	*p =* 0.002*	*p =* 0.051	-
	RFD	*p <* 0.001*	*p <* 0.001*	*p <* 0.001*	*p <* 0.001*	*p <* 0.001*	*p =* 0.008*	
	TP-EMG^a^	*p <* 0.001*	*p <* 0.001*	*p <* 0.001*	*p <* 0.001*	*p <* 0.001*	*p =* 0.496	
	EMG Duration^a^	*p <* 0.001*	*p <* 0.001*	*p <* 0.001*	*p <* 0.001*	*p =* 0.003*	*p =* 0.043*	
	RED^a^	*p <* 0.001*	*p <* 0.001*	*p <* 0.001*	*p =* 0.015*	*p =* 0.026*	*p =* 0.286	
	Peak EMG^a^	*p <* 0.001*	*p <* 0.001*	*p <* 0.001*	*p <* 0.001*	*p <* 0.001*	*p =* 0.811	

Note. Benjamini-Hochberg procedure was used to control the false discovery rate (FDR) at the level of significance, α = 0.05. ^a^ Wilcoxon Signed-Rank Tests.

### TPF and RFD Associations with EMG

Using a stepwise multiple linear regression model (Figure 6), we found that lengthened TPF at 2% MVC relative to 15% MVC significantly associated (R^2^ = 0.72, DW = 1.76, *p*< 0.05) with lengthened TP-EMG (part r = 0.63) and the increase in the variability (CV) of EMG Duration (part r = −0.33). However, the increase in TPF at 85% MVC relative to 30% MVC significantly associated (R^2^ = 0.84, DW = 1.60, *p*< 0.01) with the increase in TP-EMG (part r = 0.47) and the increase in iEMG (part r = 0.32). For PF, the increase in PF from 2 to 15% MVC significantly associated (R^2^ = 0.24, DW = 2.75, *p*< 0.05) with the increase in the variability (CV) of iEMG (part r = 0.49). However, the increase in PF from 30 to 85% MVC significantly associated (R^2^ = 0.31, DW = 2.40, *p*<0.05) with the increase in iEMG-TP (part r = 0.56).

### Clustering

The RFD slope up to the IP (initial slope) varied across our 18 participants (Figure 4C). A K-means cluster analysis identified two distinct groups (Figure 7A). Half of the participants (N = 9) exhibited slow rates for the initial RFD slope (termed Slow Initial) and the other half of the participants (N = 9) exhibited fast rates for the initial RFD slope (termed Fast Initial). We found that the overall RFD slope was significantly correlated with the initial RFD slope (R^2^ = 0.77, *p*< 0.001; Figure 7B) and the terminal RFD slope (R^2^ = 0.71, *p*< 0.001), suggesting that the RFD at effort levels below 30% MVC can predict the whole RFD slope.

### FDI Muscle Activity

The repeated measures two-way ANOVA showed that there was a main effect on the CV of iEMG (Figure 7C) between two clustering groups, Slow Initial and Fast Initial, at four low effort levels (2, 5, 15, and 30% MVC). We found that the CV of iEMG was significantly greater (F(1,16) = 7.3, *p*< 0.05, η^2^ = 0.3) for the Fast Initial group (28.1%) than the Slow Initial group (15.0%). Participants in the Fast Initial group exhibited a significantly greater CV of iEMG suggesting greater variability of iEMG in that group. The independent *t*-test showed that there was a difference in the EMG modulation between Slow Initial and Fast Initial groups. We observed that the 13–30:30–60 Hz normalized power ratio was significantly lower (t(16) = 2.2, *p*< 0.05, Cohen’s *d* = 0.9) for the Fast Initial group (ratio = 2.0) than the Slow Initial group (ratio = 2.9). Participants in the Fast Initial group exhibited a significantly lower ratio suggesting lesser power from 13–30 Hz relative to 30–60 Hz.

## Discussion

The purpose of this study was to test the speed-control hypothesis during ballistic contractions by examining effort levels from 2 to 85% MVC. For the first time in the literature, we tested the speed-control hypothesis during ballistic contractions with effort levels below 20% MVC and showed that low effort levels had distinct profiles. In contrast to the assumption by the speed-control hypothesis of constant TPF with effort, we found that TPF varied (non-linearly) with effort. The shortest TPF occurred at 15% MVC. TPF at 2% MVC was longer than TPF at 15% MVC. Furthermore, we found that the RFD increase with effort was faster below 30% MVC (initial slope) than at effort levels ranging from 30 to 85% MVC. The muscle activity findings showed that the RFD at low effort levels was influenced by neuronal variability, whereas the RFD at moderate-to-high effort levels was influenced by neuronal amplitude. We found that there were two distinct groups of participants in terms of their RFD. Half exhibited a faster slope of the increase in the RFD to 30% MVC, and also exhibited significantly lower 13–30:30–60 Hz EMG power. These findings challenge the speed-control hypothesis and provide evidence that ballistic contractions with effort levels below 30% MVC have distinct RFD profiles and muscle activity suggesting unique neural mechanisms controlling them.

### Speed of Ballistic Contractions at Low Effort Levels

The speed-control hypothesis developed by Freund and Büdingen (1978) proposes that the rate of force development (RFD) during ballistic contractions is dictated by the force amplitude. Although this hypothesis is supported by many studies, it is limited by testing effort levels above 20% MVC. For example, there was a strong linear relationship between peak force and the corresponding RFD during a set of rapid isometric muscular contractions above 20% MVC (Bellumori et al., 2011). This linear relationship was accompanied by the consistent times to reach peak force between 20 and 100% MVC (Kozinc et al., 2022). However, to our knowledge, no study has tested the speed-control hypothesis at low effort levels, which are characterized by unique motor control. In the past 40 years, there has been robust evidence that low effort ballistic contractions are more variable than moderate and high effort ballistic contractions (Newell and Carlton, 1985). This finding has been shown in young and older adults (Christou and Carlton, 2001, 2002; Enoka et al., 2003), as well as for the upper and the lower limb (Christou et al., 2010; Kwon et al., 2011; Ofori et al., 2018), suggesting unique neural control of low effort levels. Here, we provide novel evidence challenging the speed-control hypothesis, which proposes constant TPF, constant EMG duration, and a constant increase in the RFD with the effort level. Below, we provide findings that contrast the tenets of the speed-control hypothesis.

### TPF Is Not Constant but Varies with Effort

According to the speed-control hypothesis, TPF remains approximately constant with the effort level (Bellumori et al., 2011; Boccia et al., 2018; Freund and Büdingen, 1978; Kozinc et al., 2022). In contrast, we found that the relationship between TPF and the effort level was a non-linear U-shape and TPF was minimum at 15% MVC (Figure 4A). At effort levels above 15% MVC, TPF increased with the effort level. Lengthened TPF at 2% MVC relative to 15% MVC was associated with increased variability of EMG Duration, while the increase in TPF from 30 to 85% MVC was associated with increases in iEMG. Given the increased muscle activity variability associated with longer TPF at low effort levels, the speed of low-effort ballistic contractions may be influenced by the inherently greater trial-to-trial variability of the neural drive. In contrast, the lengthening of TPF with the effort level above 30% MVC was associated with the amplitude of the neural drive. These findings do not support the speed-control hypothesis for effort levels below 30% MVC.

### RFD Increases at a Faster Rate across Low Effort Levels

A major assumption of the speed-control hypothesis is that the RFD during ballistic contractions increases consistently with the effort level (Bellumori et al., 2011; Boccia et al., 2018; Freund and Büdingen, 1978). Although we found that the RFD increased with the effort level (Figure 4B, Table 1), there is a clear inflection point (IP) on the RFD curve (28.1% MVC) that indicated two distinct slopes (Figure 2B). The first slope, which is faster, occurred from 2 to 30% MVC, and the second (slower) slope occurred from 30 to 85% MVC (Figure 4). These findings are further supported by our EMG measures showing a unique neural activation of muscle controlling the force output for the two slopes. Specifically, the RFD below 30% MVC was associated with neuronal variability (CV of EMG), whereas the RFD above 30% MVC was associated with the neuronal amplitude (EMG impulse). We found that increased variability of the iEMG and increased variability of EMG Duration contributed to the increased RFD at low effort levels, however, the increment of the RFD at high effort levels was modulated by the increase in impulse of EMG. This finding suggests that the RFD at low effort levels is influenced by neuronal variability, whereas the RFD at higher effort levels is influenced by neuronal amplitude.

### RFD Slope at Low Effort Levels Is Informative

All previous studies focused on the RFD using effort levels above 20% MVC. Here, we used effort levels ranging from 2 to 85% MVC and found that the RFD increased at a greater rate for effort levels below 30% MVC. First, we found that the slope of the RFD at low effort levels could predict the total slope (Figure 7B). The overall RFD slope correlated more strongly with the initial RFD slope (R^2^ = 0.77) than the terminal RFD slope (R^2^ = 0.71). The RFD slope at effort levels below 30% can be an indicator to anticipate the whole RFD profile. Second, we found that there were two distinct types of participants in terms of the RFD slope (Fast Initial and Slow Initial), who were identified based on their RFD below 30% MVC (Figure 7A). There was greater variability (CV) of iEMG in the Fast Initial group than the Slow Initial group (Figure 7C), which indicates that the variability of iEMG is a factor that modulates the RFD slope during low-effort ballistic contractions.

### Neuronal Activity Is Unique at Low Effort Levels

We demonstrated different neural oscillations between faster and slower participants, highlighting unique neuronal activity during low-effort ballistic contractions. Faster participants exhibited a reduced ratio of normalized power in beta (13–30 Hz) to gamma (30–60 Hz) oscillations (Figure 7D). This finding provides evidence that the ratio of beta and gamma oscillation has speed-related modulations. Beta oscillations are associated with an anti-kinetic function such as movement inhibition and steadiness in voluntary movements (Brown, 2000; Delmas et al., 2018; Engel and Fries, 2010; Neto et al., 2010). However, the independent function of beta oscillations and the characteristics of ballistic contractions were not compatible, as participants were instructed to exert targeted force in a brief time (< 220 ms) without any chance of motion inhibition or steadiness. Our results align with previous evidence showing that the duration and speed of ballistic finger contractions were not related to the independent power of beta oscillations (Pfurtscheller et al., 1998). Gamma oscillations reflect the level of sensorimotor processing and execution (Aoki et al., 1999; Jensen and Mazaheri, 2010), increasing in accordance with targeted force amplitude. During ballistic contractions, the independent function of gamma oscillations and the force amplitude remain compatible across the entire effort level range. Consequently, participants with a faster RFD slope may exhibit relatively decreased beta oscillation and increased gamma oscillation, resulting in a lower beta:gamma ratio in muscle activity during low-effort ballistic contractions. Participants with a slower RFD initial slope might prioritize precise control to minimize the variability, while those with a faster RFD initial slope might focus more on speed generation, reflecting distinct organization of neural command. These findings imply that EMG modulation during low-effort ballistic contractions involves a distinct structure of the neural oscillation to simultaneously achieve two inherent goals of ballistic contractions, force amplitude and time.

## Limitations

This research presents several limitations to be considered. This study is limited to measures from a single-joint movement. Future studies should measure the speed-related modulation of low-force ballistic contractions using multiple-joint movements to generalize our findings. Another limitation is that our findings cannot be generalized to other age groups or individuals with movement disorders. Finally, future studies should record activity from higher centers using electroencephalographic recording (EEG) or magnetic resonance imaging (MRI), and examine how these measures differ during low-force ballistic contractions from moderate-to-high force levels of ballistic contractions.

## Conclusions

For the first time in the literature, the speed-control hypothesis was tested at very low effort levels below 20% MVC. The findings from this study challenge the speed-control hypothesis and provide that the RFD below 30% MVC is influenced primarily from neuronal variability, whereas the RFD above 30% MVC is primarily influenced by the amplitude of the neural drive. Our findings suggest that the regulation of speed control for low and high effort levels was clearly different, emphasizing the necessity for a new or a revised theoretical framework to understand the whole effort range of ballistic contractions.

## ORDIC iD:

Joongsuk J. Kim: 0000-0002-9630-7925

Stefan Delmas: 0000-0002-0054-3908

Yoon Jin Choi: 0000-0003-3982-3728

Jessica C. Hubbard: 0009-0004-1235-7290

BasmaYacoubi: 0000-0002-1929-4817

Evangelos A. Christou: 0000-0003-4890-4706
